# Reprogramming lipid metabolism in pediatric cancers

**DOI:** 10.1038/s41389-026-00617-1

**Published:** 2026-04-30

**Authors:** Vinzent L. Lindemann, Nazek Noureddine, Raphael J. Morscher

**Affiliations:** 1 Pediatric Cancer Metabolism Laboratory, Children’s Research Center, University of Zurich, Zurich, Switzerland; 2 Division of Oncology, University Children’s Hospital Zurich and Children’s Research Center, University of Zurich, Zurich, Switzerland

**Keywords:** Cancer metabolism, Targeted therapies, Embryonal neoplasms, Paediatric cancer, Cell growth

## Abstract

Metabolic reprogramming is a defining feature of malignant transformation and cancer cell growth. Pediatric cancers arise from genetic disruptions hijacking developmental programs by aberrant transcriptional networks. This coordinated rewiring shapes lipid metabolism through activation of biosynthetic pathways, membrane remodeling, and metabolic flexibility. This review synthesizes recent advances in the understanding of lipid metabolism reprogramming across pediatric cancers, examining four key areas: (1) transcriptional drivers that activate fatty acid and cholesterol synthesis; (2) lipid catabolism sustaining ATP, acetyl-CoA and NADPH pools under metabolic stress; (3) ferroptosis evasion through desaturation pathways and membrane remodeling; and (4) tissue-specific metabolic adaptations enabling metastasis to the bone marrow and cerebrospinal fluid. Despite extensive preclinical evidence identifying targetable vulnerabilities – including dependencies on FASN, SCD, and HMGCR – clinical impact remains to be proven. We discuss challenges of introducing therapies targeting lipid metabolism to the clinic and argue that the future lies in a better understanding of lipid flux and patient-specific dependencies.

## Introduction

Children are not small adults, nor do childhood cancers mirror adult cancers. Pediatric cancers arise from genetic disruptions, often structural alterations [1], that hijack developmental programs [2]. Through reactivation or disruption of developmentally restricted transcription factors, pediatric cancers sustain elevated proliferation [2, 3]. Such programs of transcriptional networks also rewire metabolic pathways to support physiological cell growth and differentiation [4]. In contrast, adult cancers evolve in the context of aging and maintenance-oriented metabolism, emerging after years of environmental exposure and the gradual accumulation of somatic mutations [5, 6].

Throughout development lipid metabolism is precisely regulated [7, 8]. This reflects the critical role of lipids beyond merely being structural components of membranes and energy reserves, but also functioning as regulators of ferroptosis [9], substrates for protein modification [10] and signaling molecules [11]. Pediatric cancers exploit these functions through transcriptional programs that coordinately activate lipid biosynthesis [12], remodel membrane composition [13] and tune catabolic flexibility [14, 15].

Despite mounting preclinical evidence of actionable vulnerabilities, therapies targeting lipid pathways in cancers are still lacking in the clinics. In this review, we synthesize recent advances connecting lipid metabolism to pediatric cancer pathogenesis and highlight unanswered questions that define priorities for future research. We then discuss recent advances in lipid metabolism-targeted therapies, evaluate the potential of dietary interventions, and propose a framework for advancing the field.

## Lipid pathways at a glance

The interconnected and multifunctional nature of lipid metabolism makes it challenging to fully understand the consequences of reprogramming in cancer. We therefore outline the core lipid pathways below, organized by their functions most relevant to pediatric cancer biology.

### Biosynthetic pathways: building blocks and signaling

**Fig. 1:**
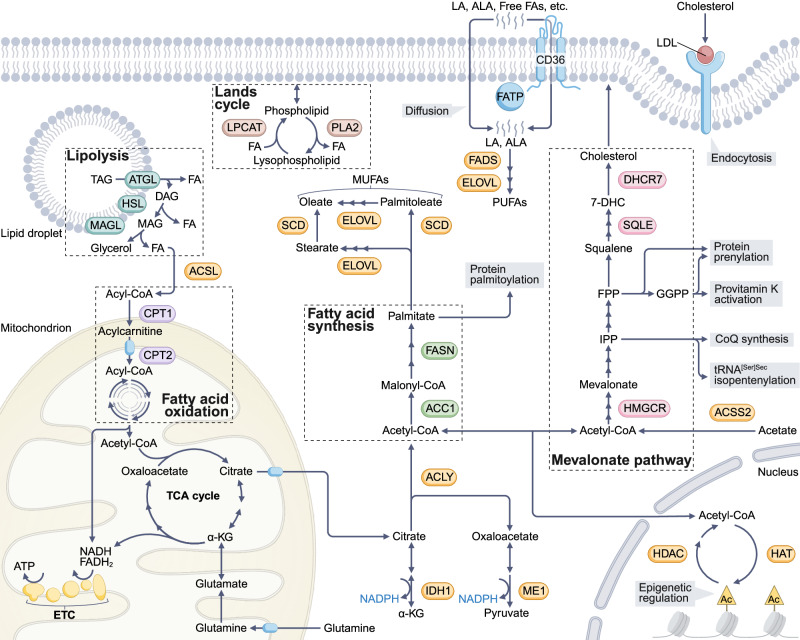
Major lipid metabolism pathways. *Fatty acid synthesis* produces palmitate, which serves as a precursor for SFAs and MUFAs. Additionally, palmitate fuels protein palmitoylation, a covalent modification that anchors proteins in membranes and plays an important role in signaling and protein trafficking. The *mevalonate pathway* generates isoprenoids used for tRNA isopentenylation, CoQ synthesis, provitamin K activation, and protein prenylation, as well as cholesterol, which is incorporated into cell membranes. Cholesterol can also be obtained through the receptor-mediated endocytosis of LDL. Extracellular free FAs and the essential PUFAs LA and ALA enter cells via passive diffusion and the membrane transport protein CD36. FATPs facilitate the uptake of long-chain FAs by enhancing their conversion to acyl-CoAs. The *Lands cycle* modifies the FA composition of phospholipids in membranes. Lipid droplets store TAG, which are hydrolyzed through *lipolysis* to release FAs when energy demand rises. These FAs enter *fatty acid oxidation* (FAO), providing reducing equivalents for ATP production and acetyl-CoA, which is condensed with oxaloacetate to form citrate in the TCA cycle. Under specific conditions, mitochondrial citrate is also produced from glutamine via glutaminolysis and reductive carboxylation. Mitochondrial citrate can be exported to the cytosol, where it is converted to acetyl-CoA through ACLY and supports NADPH production through IDH1 or ME1. Cytosolic acetyl-CoA, in addition to fueling FAS and the mevalonate pathway, can also diffuse into the nucleus, where it contributes to epigenetic regulation through histone acetylation. α-KG, a-ketoglutarate; ACC, acetyl-CoA carboxylase 1; ACLY, ATP citrate lyase; ACSL, acyl-CoA synthetase long-chain family member; ACSS2, acyl-CoA synthetase short-chain family member 2; ALA, α-linolenic acid; ATGL, adipose triglyceride lipase; ATP, adenosine triphosphate; CD36, fatty acid translocase; CoQ, coenzyme Q10; CPT1, carnitine palmitoyltransferase 1; CPT2, carnitine palmitoyltransferase 2; DAG, diacylglycerol; DHCR7, 7-dehydrocholesterol reductase; ELOVL, fatty acid elongase; ETC, electron transport chain; FA, fatty acid; FADH_2_, reduced flavin adenine dinucleotide; FADS, fatty acid desaturase; FASN, fatty acid synthase; FATP, fatty acid transport protein; FPP, farnesyl diphosphate; GGPP, geranylgeranyl diphosphate; HAT, histone acetyltransferase; HDAC, histone deacetylase; HMGCR, 3-hydroxy-3-methylglutaryl-CoA reductase; HSL, hormone-sensitive lipase; IDH1, isocitrate dehydrogenase 1; IPP, isopentenyl diphosphate; LA, linoleic acid; LDL, low-density lipoprotein; LPCAT, lysophosphatidylcholine acyltransferase; MAG, monoacylglycerol; MAGL, monoacylglycerol lipase; ME1, malic enzyme 1; MUFAs, monounsaturated fatty acids; NADH, reduced nicotinamide adenine dinucleotide; NADPH, reduced nicotinamide adenine dinucleotide phosphate; PLA2, phospholipase A2; PUFAs, polyunsaturated fatty acids; SCD, stearoyl-CoA desaturase; SQLE, squalene epoxidase; TAG, triacylglycerol; TCA Cycle, tricarboxylic acid cycle; 7-DHC, 7-dehydrocholesterol. For simplicity, stoichiometry of reactions is not indicated. Created in BioRender. Lindemann, V. (2026) https://BioRender.com/y9y43s0.

*Fatty acid synthesis (FAS)* generates palmitate in the cytosol from acetyl-CoA, which is predominantly derived from citrate via ATP citrate lyase (ACLY) or from acetate via acyl-CoA synthetase short-chain family member 2 (ACSS2) [16]. Acetyl-CoA carboxylase 1 (ACC1, also known as ACACA) irreversibly converts acetyl-CoA to malonyl-CoA, which is then polymerized by fatty acid synthase (FASN) to produce palmitate (Fig. 1). Many pediatric cancers upregulate this pathway through transcriptional programs [12, 13, 15, 17], enabling sustained growth even when extracellular lipid supply is limited.

*The mevalonate pathway* produces cholesterol de novo from acetyl-CoA through a branched, multi-step process spanning the cytosol and ER. Its rate-limiting enzyme 3-hydroxy-3-methylglutaryl-coenzyme A reductase (HMGCR) generates mevalonate, which is subsequently converted into isoprenoid intermediates (Fig. 1). Besides fueling cholesterol synthesis, these isoprenoids are also used for coenzyme Q10 (CoQ) synthesis [18], conversion of menadione (provitamin K) to biologically- active menaquinone-4 [19] as well as protein prenylation, which anchors signaling proteins to membranes [20]. Downstream, squalene epoxidase (SQLE) functions as the rate-limiting enzyme of the committed cholesterol biosynthesis branch. Cholesterol itself is an essential membrane component and can facilitate oncogenic signaling by stabilizing lipid rafts, which concentrate signal transduction processes, or by directly interacting with signaling proteins [21, 22]. In addition to obtaining cholesterol via receptor-mediated endocytosis of low-density lipoprotein (LDL), cancer cells often upregulate endogenous synthesis [21, 23]. However, the numerous pathway intermediates and metabolic branches emanating from them suggest cancer-relevant functions beyond cholesterol provision alone.

### Lipid remodeling: tuning membrane composition and ferroptosis sensitivity

*Desaturation and elongation pathways* diversify the pool of fatty acids (FAs) by introducing double bonds and extending carbon chains, respectively. Sequential reactions catalyzed by fatty acid elongases (ELOVLs) and stearoyl-CoA desaturase (SCD) convert saturated fatty acids (SFAs) into monounsaturated fatty acids (MUFAs). Polyunsaturated fatty acids (PUFAs) cannot be synthesized endogenously, and the essential PUFAs linoleic acid (LA) and α-linolenic acid (ALA) must be obtained through diet [24]. Successive reactions catalyzed by ELOVLs and fatty acid desaturases (FADSs) generate longer and highly unsaturated PUFAs, such as arachidonic acid and docosahexaenoic acid. Cells use this rich FA pool in part to produce complex lipids.

*Phospholipid remodeling* enables cells to generate new phospholipids without synthesizing them entirely de novo. A central pathway is the Lands cycle, which modifies the FA composition of glycerophospholipids. Mechanistically, phospholipase A2 (PLA2) removes an esterified FA, and re-acylation enzymes such as lysophosphatidylcholine acyltransferases (LPCATs) then incorporate an alternative FA (Fig. 1). Cancer cells may exploit this process and preferentially incorporate SFAs or MUFAs to escape ferroptosis, a PUFA-dependent form of cell death [25].

### Lipid catabolism: energy and stress buffering

*Fatty acid oxidation (FAO)* catabolizes FAs in mitochondria through iterative β-oxidation cycles. Long-chain FAs must first be activated to acyl-CoAs by acyl-CoA synthetase long-chain family members (ACSLs) prior to mitochondrial import via the carnitine shuttle (Fig. 1). Carnitine palmitoyltransferase 1 (CPT1) catalyzes the rate-limiting step of FAO. Short-chain FAs can diffuse into the mitochondrial matrix before they are activated to their CoA-derivatives for catabolic use. Each subsequent β-oxidation cycle shortens the FA by two carbons, yielding acetyl-CoA for the TCA cycle as well as NADH and FADH₂, which directly feed into the electron transport chain (ETC) to generate ATP (Fig. 1).

*Lipid droplets* store triacylglycerols (TAGs) as well as cholesteryl esters and function as metabolic stress buffers. The precursors of TAGs and cholesteryl esters are bioactive lipids that disrupt normal cell function when present in excess (lipotoxicity) [26]. By absorbing free FAs in particular, lipid droplets alleviate ER stress [26] and the sequestration of peroxidation-prone PUFAs further protects cells from oxidative stress [9]. During energy demand, lipolysis or lipophagy, the selective autophagy of lipid droplets, mobilizes stored FAs and fuels FAO [27]. These mechanisms explain why the frequently observed accumulation of lipid droplets functions as a versatile survival strategy in cancer [14, 26].

## Transcriptional programs drive lipid biosynthesis

In pediatric cancers, major genetic events such as chromosomal translocations (e.g. *EWSR1::FLI1, PAX3::FOXO1*) or gene amplifications (e.g. *MYCN*) trigger the formation of transcriptional programs [12, 28, 29]. Despite the molecular diversity, these programs often converge on the activation of key lipid pathways: FAS [15, 17], MUFA synthesis [13, 17] and the mevalonate pathway [23, 30, 31].

### Reprogramming of fatty acid synthesis

**Fig. 2:**
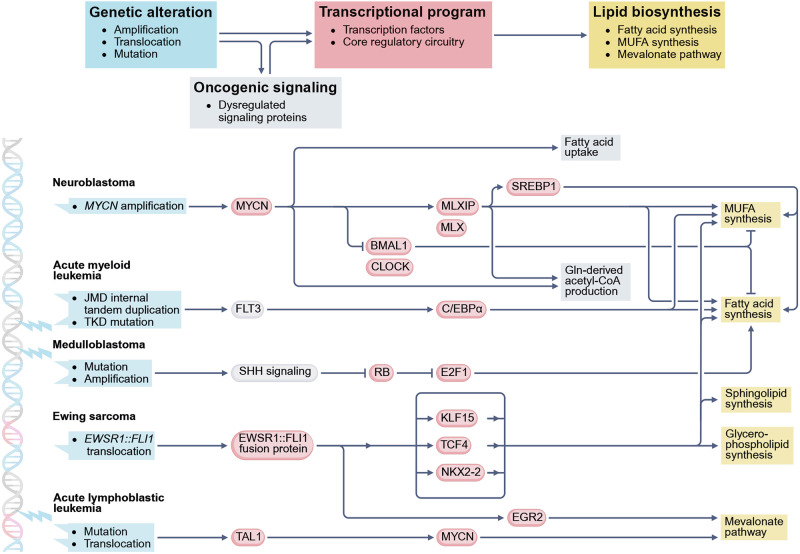
Molecular cascades establish lipogenic transcriptional programs. Oncogenic programs are induced by genetic alterations (highlighted in blue), that are characteristic of specific pediatric cancer types and give rise to fusion proteins or dysregulate master transcription factors and signaling pathways. The resulting transcriptional programs (highlighted in red) coordinately upregulate enzymes of various lipid pathways (highlighted in yellow). This cascade provides essential lipids for cancer cell growth and survival. BMAL1, basic helix-loop-helix ARNT like protein 1; C/EBPα, CCAAT-enhancer-binding protein α; CLOCK, circadian locomoter output cycles protein kaput; EGR2, early growth response 2; FLT3, Fms-like tyrosine kinase 3; Gln, glutamine; JMD, juxta-membrane domain; KLF15, Kruppel-like factor 15; MLX, Max-like protein X; MLXIP, MLX-interacting protein; MUFA, monounsaturated fatty acid; NKX2-2, homeobox protein Nkx2.2; RB, retinoblastoma protein; SHH, Sonic hedgehog; SREBP1, sterol regulatory element-binding protein 1; TAL1, T-cell acute lymphocytic leukemia protein 1; TCF4, transcription factor 4; TKD, tyrosine kinase domain. Created in BioRender. Lindemann, V. (2026) https://BioRender.com/igr5wmz.

Distinct transcriptional programs drive FAS across pediatric cancers (Fig. 2). In high-risk neuroblastoma, a multi-layered transcriptional program driven by *MYCN* amplification activates FAS [17, 32]. MYCN upregulates the transcription factor MLX-interacting protein (MLXIP, formerly known as MondoA) and cooperates with the MLXIP-MLX dimer to funnel glutamine into the TCA cycle [17]. Subsequent reductive carboxylation produces citrate, which is exported to the cytosol and converted to acetyl-CoA to fuel FAS (Fig. 1) [17]. MLXIP-MLX directly induces FASN and SCD and indirectly reinforces FAS via sterol regulatory element-binding protein 1 (SREBP1) [17], forming a feedforward loop [33]. MYCN also represses the molecular circadian clock, overriding its inhibitory effect on lipogenesis [32], and directly upregulates long-chain fatty acid transport protein 2 (FATP2) to enhance FA uptake [34]. Taken together, this metabolic reprograming secures the fatty acid supply necessary for neuroblastoma proliferation through both biosynthesis and uptake.

In acute myeloid leukemia (AML), constitutively active mutant Fms-like tyrosine kinase 3 (FLT3) phosphorylates CCAAT-enhancer-binding protein α (C/EBPα), which in turn induces FASN and SCD [13]. While this reprogramming funnels FASN-produced palmitate into desaturation pathways catalyzed by SCD [13], palmitate is also used for S-palmitoylation of FLT3 [35]. This covalent modification retains the mutant kinase in the ER and alters downstream signaling [35, 36]. Although these studies provide mechanistic insights into FLT3-driven lipid metabolism, their relevance to pediatric AML has yet to be evaluated in age-defined cohorts. This is particularly important given evidence that FLT3 mutations exert developmental context-specific effects on myeloid leukemogenesis [37].

In Sonic hedgehog medulloblastoma (SHH MB), developmental SHH signaling is exploited to maintain cancer cell growth through FASN upregulation [15]. This appears to be mediated by SHH-driven phosphorylation of the retinoblastoma protein (RB), which permits activation of the transcription factor E2F1 [15]. Importantly, treatment of tumor-bearing mice with a FASN inhibitor prolonged survival, underscoring the dependence of SHH MB on intrinsic lipid synthesis [15]. The clinical relevance of this dependence is further supported by the observation that FASN and SCD expression levels are prognostic in three of four MB subgroups defined by the World Health Organization (SHH group, group 3, group 4) [38].

In Ewing sarcoma the oncogenic fusion protein EWSR1::FLI1 establishes super-enhancers for three transcription factors regulating metabolism: KLF15, TCF4 and NKX2-2 [12]. These factors co-bind to their own and each other’s super-enhancers, amplifying their activity through positive feedback [12]. This so-called core regulatory circuitry coordinately induces enzymes for the synthesis of FAs, MUFAs, sphingolipids and glycerophospholipids (Fig. 2), suggesting that Ewing sarcoma thrives on synchronized reprogramming of multiple lipid pathways [12]. Moreover, EWSR1::FLI1 directly activates the transcription factor early growth response 2 (EGR2), which upregulates the mevalonate pathway [39].

Like FAS, the mevalonate pathway is activated in many pediatric cancers [23, 30, 31, 39–41]. Given that both pathways provide essential lipid building blocks, this raises the question of whether they serve distinct or overlapping functions.

### Reprogramming of the mevalonate pathway

**Fig. 3:**
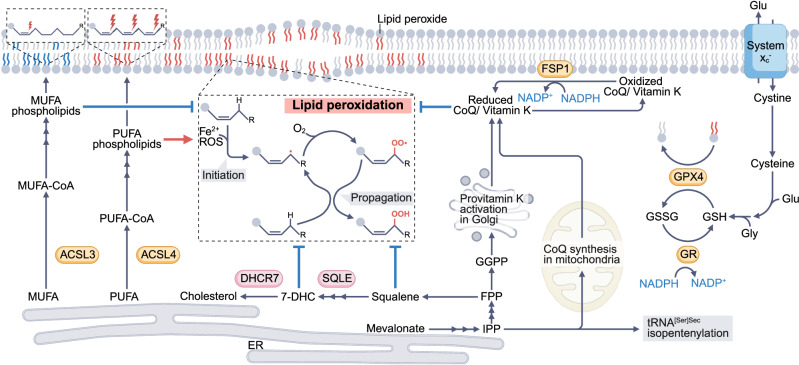
Regulation of ferroptosis sensitivity in pediatric cancers. Peroxidation of PUFA-containing phospholipids is initiated by reactive oxygen species (ROS), which can arise from the iron-catalyzed Fenton reaction. Lipid radicals (L⋅) then react with oxygen to form lipid peroxyl radicals (L-OO⋅), which in turn react with neighboring PUFA-containing phospholipids, thereby producing lipid hydroperoxides (L-OOH) as well as new lipid radicals. This propagation is amplified by iron, which catalyzes the decomposition of hydroperoxides into lipid hydroxyl and lipid alkoxyl radicals. The accumulation of altered lipid species damages membranes and may ultimately lead to ferroptosis if not counteracted. Pediatric cancers utilize numerous mechanisms to escape this form of cell death. GPX4, GR and system x_c_^-^ work in conjunction to deplete lipid hydroperoxides through reduction. Parallel to this canonical protection, FSP1 regenerates the reduced forms of CoQ and vitamin K, which stop lipid peroxidation by donating electrons to lipid radicals. These antioxidants are synthesized or activated, respectively, through isoprenoid intermediates from the mevalonate pathway. Alongside this, IPP can also be attached to the selenocysteine tRNA as an isopentenyl moiety, promoting translation of the selenoprotein GPX4. The mevalonate pathway is closely linked with the regulation of ferroptosis sensitivity and further provides the antioxidant 7-DHC and squalene. While both molecules suppress ferroptosis, the mechanism for squalene’s effect is incompletely understood. Lastly, ACSL3-mediated MUFA incorporation into phospholipids promotes ferroptosis resistance, whereas ACSL4-mediated PUFA incorporation increases ferroptosis sensitivity. ACSL3, acyl-CoA synthetase long-chain family member 3; ACSL4, acyl-CoA synthetase long-chain family member 4; CoQ, coenzyme Q10; DHCR7, 7-dehydrocholesterol reductase; ER, endoplasmic reticulum; FPP, farnesyl diphosphate; FSP1, ferroptosis suppressor protein 1; GGPP, geranylgeranyl diphosphate; Glu, glutamate; Gly, glycine; GPX4, glutathione peroxidase 4; GR, glutathione reductase; GSH, reduced glutathione; GSSG, oxidized glutathione; IPP, isopentenyl diphosphate; MUFA, monounsaturated fatty acids; NADP^+^, oxidized nicotinamide adenine dinucleotide phosphate; NADPH, reduced nicotinamide adenine dinucleotide phosphate; PUFA, polyunsaturated fatty acids; ROS, reactive oxygen species; SQLE, squalene epoxidase; System x_c_^-^, cystine/glutamate transporter; 7-DHC, 7-dehydrocholesterol. Created in BioRender. Lindemann, V. (2026) https://BioRender.com/luq61dh.

The rationale underlying activation of the mevalonate pathway remains incompletely understood. In TAL1-positive T-cell acute lymphoblastic leukemia (T-ALL), ectopically expressed TAL1 activates a *MYCN* enhancer, which enables MYCN to induce multiple genes involved in the mevalonate pathway [23]. This transcriptional rewiring is essential, as TAL1-positive T-ALL cells are highly sensitive to HMGCR inhibition [23]. Notably, cholesterol supplementation does not rescue cell viability, whereas geranylgeranyl diphosphate (GGPP) – an isoprenoid intermediate from the non-sterol branch of the pathway (Fig. 1) – partially restores survival [23]. GGPP is a substrate for protein prenylation [20] and provitamin K activation [19], highlighting roles of the mevalonate pathway in signaling and ferroptosis protection (Fig. 3). However, the mechanism underlying the survival advantage of mevalonate pathway activation in TAL1-positive T-ALL cells remains poorly defined.

Early T-cell precursor ALL (ETP-ALL) is similarly sensitive to HMGCR inhibition, which was shown to disrupt AKT1 (AKT serine/threonine kinase 1) signaling in this setting [42]. Here, cholesterol or GGPP supplementation fully rescues viability and signaling [42]. As AKT1 localizes to cholesterol-rich lipid rafts, it is particularly sensitive to changes in membrane cholesterol, which may explain part of the rescue phenotype [42, 43]. Nonetheless, the role of GGPP and thus how the mevalonate pathway exactly sustains AKT1 signaling in ETP-ALL remains unclear.

The link between oncogenic signaling and the mevalonate pathway is better defined in SHH MB. Here, membrane sterols directly bind and activate the signaling protein smoothened (SMO) [22, 44, 45]. Although upstream reprogramming is yet to be determined, the mevalonate pathway genes are highly expressed in SHH MB compared to all other MB subgroups [46]. This suggests a positive feedback loop whereby SHH-driven reprogramming increases membrane sterol production, which in turn amplifies oncogenic signaling [22, 44–46].

Together, these examples illustrate that activation of the mevalonate pathway does not necessarily only serve provision of cholesterol as a building block. Instead, it also supports protein prenylation in TAL1-positive T-ALL [23] and ETP-ALL [42] as well as oncogenic signaling in SHH MB [22, 44–46]. Which of these mechanisms predominates in different pediatric cancers, and whether they can be targeted specifically, remains an open question.

Stepping back, unifying principles of lipid metabolism reprogramming become apparent. First, transcriptional programs actively drive lipid biosynthesis rather than passively responding to nutrient scarcity. Second, diverse transcriptional programs converge on common metabolic outputs: FAS [15, 17], MUFA synthesis [13, 17] and the mevalonate pathway [23, 30, 31]. Third, upregulated lipid pathways serve different purposes depending on the cellular context. What advantages justify this conserved metabolic rewiring? The answer extends beyond building blocks: reprogramming lipid metabolism amplifies oncogenic signaling [22, 44–46], supports catabolic flexibility during stress [47], enables ferroptosis escape [13] and facilitates metastatic colonization of distinct tissue niches [48, 49]. These pleiotropic functions, discussed in later sections, help explain the consistent rewiring of lipid metabolism and why its therapeutic disruption is of high interest.

## The lipid catabolism enigma in cancer

It is increasingly recognized that cancer cells simultaneously engage in both FAS and FAO, challenging the conventional dogma of their mutual exclusivity [47]. Labeling this co-occurrence as a futile cycle is inadequate, given that FAO inhibition reduces cell proliferation across pediatric cancers, including ALL [50], AML [50, 51], rhabdomyosarcoma [52], osteosarcoma [53] and *MYCN*-amplified neuroblastoma [54]. But how do pediatric cancer cells promote FAO and for what reasons?

### Reprogramming of fatty acid oxidation

Unlike lipid biosynthesis, the transcriptional networks regulating FAO in pediatric cancers remain poorly defined. In principle, core oncogenic programs coordinate both anabolic and catabolic lipid pathways [14, 15, 17]. For example, MYCN inhibition in neuroblastoma impairs FAO [14], suggesting a role for MYCN in FAO stimulation beyond promoting FAS [17, 32]. Conversely, the SHH/E2F1 axis in medulloblastoma suppresses FAO while promoting FAS [15].

Additional regulatory mechanisms complement core transcriptional programs. In osteosarcoma, the RNA cytosine C(5)-methyltransferase (NSUN2) stabilizes fatty acid-binding protein 5 (FABP5) mRNA, thereby increasing FAO and lipolysis [53]. Burkitt lymphoma cells upregulate lipid catabolism through calcium/calmodulin-dependent protein kinase type II subunit delta (CAMK2D) [55]. This has been suggested to result from destabilization of transcription factor forkhead box protein O3 (FOXO3A) and consequent transcription of downstream interferon regulatory factor 4 (IRF4) [55]. Yet, while IRF4 promotes lipolysis in adipocytes [56], its role in regulating lipid metabolism in Burkitt lymphoma remains to be experimentally validated [55]. These examples illustrate that FAO is controlled at multiple levels, however a comprehensive understanding of these regulatory networks is lacking.

### Functions of fatty acid oxidation in cancer

*Energy generation* is the primary function of FAO. Degradation of a single palmitate yields remarkable ~106 molecules of ATP if the resulting acetyl-CoA is catabolized in the TCA cycle and reducing equivalents are funneled into the ETC (Fig. 1). This highlights the importance of mitochondrial respiration in cancer alongside enhanced aerobic glycolysis (the Warburg effect), as many cancers exploit both bioenergetic pathways [54, 57]. In doing so, malignant cells gain metabolic flexibility, enabling them to survive even as nutrient availability fluctuates.

*Epigenetic dysregulation* plays pro-tumorigenic roles and FAO has recently emerged as an important source for the nuclear substrate acetyl-CoA [16, 58]. This requires that mitochondrial FAO-derived acetyl-CoA first exits the mitochondria as citrate and then enters the nucleus, where it feeds into histone acetylation (Fig. 1) [16, 58]. In posterior fossa A (PFA) ependymoma, an epigenetically driven pediatric brain cancer [59], elevated histone H3 lysine 27 (H3K27) acetylation blocks methylation at this site and supports tumor survival [60]. Mass spectrometry profiling revealed elevated FAO in PFA tissues [60], suggesting that FAO-derived acetyl-CoA could contribute to this histone acetylation. This hypothesis is further supported by recent work in adult glioblastoma multiforme, where FAO inhibition led to reduced H3K27 acetylation [58].

*NADPH production* is supported indirectly by FAO. This involves the conversion of FAO-derived acetyl-CoA to citrate in the TCA cycle and subsequent export to the cytosol, where it can engage in NADPH production through malic enzyme 1 (ME1) or isocitrate dehydrogenase 1 (IDH1) (Fig. 1) [61]. NADPH serves as an essential electron donor needed for the regeneration of antioxidants and the biosynthesis of FAs, sterols, nucleotides and amino acids [61]. Evidence from adult cancers indicates that this metabolic linkage is particularly important during energy stress, when NADPH generation by the pentose phosphate pathway is impaired [62, 63], yet its relevance in pediatric cancers is largely unexplored.

### Open questions and future directions

Despite growing appreciation for the importance of FAO in pediatric cancers, fundamental questions remain. How do oncogenic programs coordinate FAS and FAO simultaneously, and under what conditions does one predominate? What is the relative contribution of FAO to ATP, acetyl-CoA and NADPH pools in different cancer types and metabolic states? Moreover, do specific vulnerabilities exist that could be targeted more precisely than by globally inhibiting FAO? A promising approach for addressing these questions is stable isotope tracing with labeled fatty acids, which could determine FAO flux and reveal the fate of FAO-derived carbons [64].

## Ferroptosis escape by all means

Cancer cells aim to avoid ferroptosis, a form of cell death triggered by the accumulation of peroxidized PUFA-containing phospholipids (Fig. 3) [9]. These lipid species arise from radical-driven reactions or through enzymatic mechanisms catalyzed by lipoxygenases [65]. Canonical protection involves the selenoprotein glutathione peroxidase 4 (GPX4), which converts lipid hydroperoxides into benign lipid alcohols using reduced glutathione (GSH). Thereafter, NADPH-dependent glutathione reductase (GR) regenerates GSH from oxidized glutathione (GSSG). As GSH contains cysteine, the cystine/glutamate transporter (system x_c_^-^) responsible for cystine import is another crucial component for GPX4 activity [66, 67]. Several pediatric cancers exploit this canonical machinery to escape ferroptosis, including ALL [68, 69], osteosarcoma [67] and neuroblastoma [66, 70, 71]. Additionally, cancer cells reprogram specific lipid pathways to obtain complementary or alternative protection.

### Crosstalk between lipid metabolism and ferroptosis

Reprogramming lipid metabolism can protect cancer cells from ferroptosis either by accumulating antioxidants or by remodeling phospholipids from PUFA-rich to SFA- or MUFA-rich compositions.

*Mevalonate pathway activation* increases levels of antioxidants, particularly in cancers that shut down part of the pathway to reroute intermediates. Loss of SQLE expression causes an accumulation of squalene in ALK-positive anaplastic large cell lymphoma (ALCL) [72]. While this protects ALCL cells from ferroptosis, it also renders them auxotrophic for cholesterol and thus highly dependent on cholesterol uptake [72]. In Burkitt lymphoma and neuroblastoma, 7-dehydrocholesterol reductase (*DHCR7*) mutations lead to a buildup of the antioxidant 7-dehydrocholesterol (7-DHC) [73, 74]. More broadly, the isoprenoid intermediates isopentenyl diphosphate (IPP), and GGPP, contribute to ferroptosis protection through their roles in antioxidant generation [9, 75–77]. IPP and GGPP are used for coenzyme Q10 (CoQ) synthesis [18] and conversion of menadione (provitamin K) to biologically active menaquinone-4 [19], respectively (Fig. 3). The reduced forms of these molecules act as lipophilic radical-trapping antioxidants that can be recycled by the NADPH-dependent ferroptosis suppressor protein 1 (FSP1) [75–77]. Finally, IPP also feeds the isopentenylation of selenocysteine tRNA, which is necessary for recoding UGA codons for selenocysteine insertion and thereby enables translation of selenoproteins such as GPX4 [78, 79].

*Decreasing the ratio of PUFAs to MUFAs plus SFAs in phospholipids* can be an effective protection strategy from ferroptosis [9, 80]. The ferroptosis-promoting role of PUFA-phospholipids stems from the greater number of double bonds that are susceptible to peroxidation as compared to MUFAs or SFAs, which only have one or none respectively. In *FLT3*-mutant AML, the core transcriptional program increases the synthesis of SFAs and MUFAs (Fig. 2) as well as their incorporation into phospholipids to evade ferroptosis [13]. Importantly, this membrane remodeling can also be therapeutically exploited [13, 81]. SCD inhibition restores ferroptosis sensitivity in resistant neuroblastoma cells, and co-treatment with the PUFA arachidonic acid further enhances ferroptotic cell death by increasing the ratio of PUFAs to MUFAs [81]. Of note, the cytotoxicity of PUFAs arises only after incorporation into membrane phospholipids, as free PUFAs do not drive ferroptosis.

ACSL4 conjugates PUFAs to CoA, a step required for their incorporation into phospholipids, and has an ambivalent role in cancer. While ACSL4 downregulation reduces ferroptosis sensitivity [82], ACSL4 upregulation can benefit cancer cells during metastasis by enhancing membrane fluidity, cellular invasiveness and signaling pathways, as shown in studies of adult cancers [83, 84]. In pediatric hepatoblastoma and osteosarcoma, in vivo *ACSL4* knockdown significantly reduces tumor growth, indicating a predominantly pro-tumorigenic role in these cancers [85, 86].

Although sensitizing cancer cells to ferroptosis is emerging as a promising therapeutic strategy, cancers are remarkably versatile in escaping [9]. These defensive mechanisms, however, entail trade-offs. During cancer progression, malignant cells encounter several metabolic roadblocks such as nutrient scarcity and new tissue environments that may require lipid metabolism reprogramming incompatible with ferroptosis resistance. As detailed above, ACSL4 upregulation exemplifies such a trade-off. Further investigations of these compromises during cancer progression will likely uncover targetable ferroptotic vulnerabilities.

## Lipids in metastasis

**Fig. 4:**
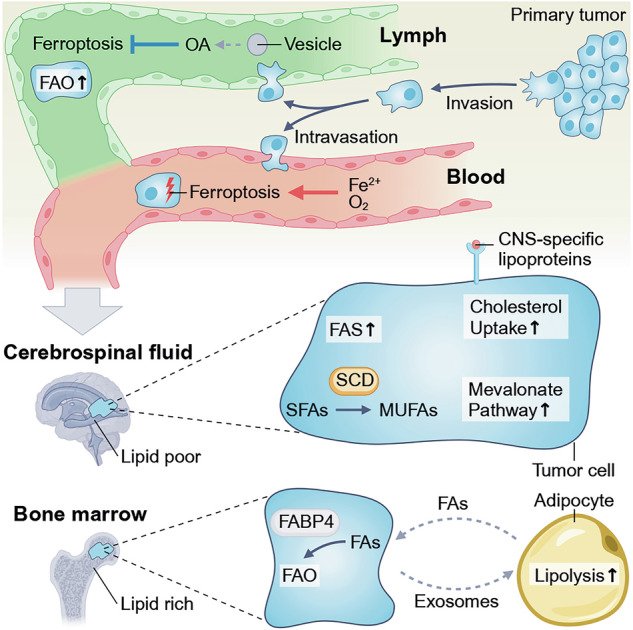
Metabolic adaptation during metastasis. The metastatic journey imposes route-specific metabolic opportunities and challenges. Vesicles storing OA in TAG can release this MUFA to protect cancer cells against ferroptosis in the lymph. Conversely, high levels of free iron and oxygen promote ferroptosis in the blood. Colonization of distant organs requires distinct metabolic adaptations with availability of lipids being a relevant driver. In the CSF, pediatric cancer cells upregulate lipid biosynthesis and uptake to compensate for the limited exogeneous supply. By contrast, the lipid-rich bone marrow harbors many adipocytes that cancer cells reprogram via exosomes to utilize their released FAs for FAO. CNS, central nervous system; FABP4, fatty acid-binding protein 4; FAO, fatty acid oxidation; FAs, fatty acids; FAS, fatty acid synthesis; FASN, fatty acid synthase; MUFAs, monounsaturated fatty acids; OA, oleic acid; SCD, stearoyl-CoA desaturase; SFAs, saturated fatty acids. Created in BioRender. Lindemann, V. (2026) https://BioRender.com/40haaom.

Metastasis requires cancer cells to navigate diverse lipid environments. Each milieu along the metastatic route and at the target niche imposes distinct metabolic opportunities and pressures. Pediatric cancer cells undergo particularly pronounced metabolic adaptations in the lipid-poor cerebrospinal fluid (CSF) [48, 49] and the lipid-rich bone marrow (Fig. 4) [87–89].

### Lipid opportunities along metastatic routes: insights from adult cancers

In lymph fluid, a lipid rich environment provides disseminating cancer cells with energy [90] and ferroptosis protection, whereas in the blood, higher levels of oxygen and free iron promote ferroptosis [91]. A study in melanoma has shown that ApoB-positive vesicles in the lymph provide the MUFA oleic acid (OA), which protects from ferroptosis in an ACSL3-dependent manner [91]. Cancer cells passing through lymph before entering the blood retain this protection, whereas those entering the bloodstream directly are more vulnerable to ferroptosis [91]. Additionally the transcriptional coactivator yes-associated protein (YAP) upregulates FAO enabling cancer cells to exploit the abundant FAs in the lymph for energy generation [90]. Whether these mechanisms also operate in pediatric cancers is not yet known.

### Adaptation to the lipid-poor cerebrospinal fluid

The CSF is extremely poor in FAs and cholesterol compared to blood plasma, forcing metastatic cells to undergo major metabolic adaptations [48, 49]. To survive in this niche, ALL cells upregulate SCD, FAS, the mevalonate pathway as well as the LDL receptor [48, 49]. A therapeutic attempt to target this vulnerability with a SCD inhibitor reduced CSF tumor burden in xenografted mice, but had little effect on bone marrow disease [48]. This demonstrates that metabolic dependencies can vary profoundly between compartments.

### The dynamic bone marrow niche

In contrast to CSF, the bone marrow is a lipid-rich environment, where metabolic crosstalk with adipocytes reduces the cancer cells’ dependence on intrinsic lipid biosynthesis [87–89]. Leukemic cells establish a bidirectional metabolic communication with bone marrow adipocytes [87–89]. Cancer-derived exosomes stimulate adipocyte lipolysis, thereby increasing the release of free FAs [87]. These FAs are then transferred back to leukemic cells via fatty acid-binding protein 4 (FABP4), where they fuel FAO to support survival and proliferation [88, 89]. In other cancer types, evidence of lipid metabolism adaptations during metastasis is lacking.

Lipid abundance in metastatic sites may also contribute to the organotropism observed in pediatric cancers. For example, high-risk neuroblastoma frequently metastasizes to the bone marrow, whereas spread to the CSF is rare [92]. This discrepancy may reflect the relative ease of colonizing a lipid-rich niche, which reduces the need for an extensive MYCN-driven lipogenic reprogramming characteristic of primary tumors (see section “Reprogramming of Fatty Acid Synthesis”). Direct evidence for this, however, is still lacking.

Beyond adipocytes, other cells in the tumor microenvironment also rewire their lipid metabolism. One such example are immune cells, in which case it directly impacts the quality of antitumor immunity [93]. Given that specific lipids have immunomodulatory functions [11, 94], this intersection between metabolism and immune response warrants further investigation.

In summary, metastatic cancer cells either rewire their intrinsic lipid metabolism [48] or exploit local lipid sources [88]. A major gap in our understanding of metabolic adaptations and organotropism across pediatric cancers stems from the scarcity of direct comparisons between primary tumors and their metastases. Moreover, how pediatric cancers engage in metabolic crosstalk with healthy organs or perturb the crosstalk among them remains uncharted.

## Targeting lipid metabolism for cancer therapy

The extensive reprogramming of lipid metabolism described throughout this review suggests multiple therapeutic opportunities, yet translating these insights into the clinics has proven challenging. So far, zero lipid metabolism targeting therapies have been clinically approved for the treatment of pediatric cancers. Nonetheless, lipid pathways remain an attractive therapeutic target, particularly within precision-medicine frameworks.

### Lipid-lowering agents

Given the frequent activation of lipid biosynthesis in pediatric cancers, different strategies have been explored to disrupt these pathways.

*FASN inhibitors* are effective in several pediatric cancer mouse models [12, 15, 95], but have not yet advanced beyond preclinical studies. While results from ongoing adult phase II trials of the FASN inhibitor TVB-2640 are still pending [NCT03808558, NCT03179904], the first positive response signals have emerged [96] and may accelerate future clinical trials in children. Similarly, targeting FA desaturation through SCD inhibition has shown preclinical effectiveness against leukemias with high pathway activity [13, 48, 97, 98]. However, development of clinical inhibitors has been hampered due to complex management of dose-limiting skin and ocular side effects [99].

*ACSS2 inhibition* is an emerging approach that targets cytosolic acetyl-CoA production upstream of lipid synthesis (Fig. 1). The first oral ACSS2 inhibitor, MTB-9655, is currently in phase I trials for advanced adult solid tumors [NCT04990739]. While preclinical studies demonstrate broad relevance of ACSS2 in cancer metabolism [100], its role in pediatric cancers remains insufficiently characterized.

*Statins* have been evaluated as potential anticancer agents given their ability to disrupt the mevalonate pathway. Although preclinical studies demonstrated promising effects in various leukemia and solid tumor entities [23, 30, 31, 39–42, 46, 101], a recent phase I trial combining simvastatin with chemotherapy for relapsed or refractory pediatric solid tumors and brain cancers reported limited therapeutic responses [102]. In this setting, drug-related toxicities may have prevented dose escalation to levels that were likely required for robust antitumor effects [102]. Beyond inhibiting lipid biosynthesis, alternative strategies target lipid catabolism and exploit ferroptosis sensitivity.

### Catabolic inhibitors and ferroptosis inducers

*CPT1 inhibition*, which blocks FAO, exerts cytotoxic effects across ALL [50], AML [50], osteosarcoma [53], and *MYCN*-amplified neuroblastoma [54]. However, CPT1’s essential role in hepatic and cardiac metabolism has raised toxicity concerns that complicate its targeting for cancer treatment [103].

*Inducing ferroptosis* represents a conceptually distinct approach that exploits, rather than blocks lipid metabolism. *MYCN*-amplified neuroblastoma cells are particularly vulnerable to this strategy as they increase iron influx, creating profound dependence on ferroptosis defense pathways such as cystine uptake and GPX4 activation (Fig. 3) [66, 70, 71]. Pharmacological inhibition of these systems resulted in tumor remission in an orthotopic *MYCN*-amplified model [66], while combining chemotherapy with a ferroptosis inducer increased survival in chemoresistant patient-derived xenografts [104]. Building on these preclinical findings, a phase I clinical trial evaluated the GSH synthesis inhibitor buthionine sulfoximine combined with melphalan for recurrent or refractory high-risk neuroblastoma [105]. The favorable tolerability observed in this trial supports continued development of ferroptosis-inducing strategies for susceptible pediatric cancers [105].

Key challenges for the clinical translation of ferroptosis-inducing strategies include the diverse tumor escape mechanisms and potential undesirable effects that could compromise antitumor immune responses. This immunological impairment may arise from the lower immunogenicity of ferroptosis compared with other forms of cell death [106] and from inducing ferroptosis in protective immune cells [107]. Future success will depend on identifying cancers that exhibit ferroptosis vulnerability and selecting appropriate therapy combinations.

The metabolic plasticity of cancer cells is a shared limitation of ferroptosis inducers and lipid metabolism inhibitors. For instance, enhanced dietary lipid uptake may compensate for blocked biosynthesis. Consistent with the importance of extracellular nutrient supply, inhibition of FA uptake exerted potent anti-cancer activity in multiple preclinical *MYCN*-amplified neuroblastoma models [34]. This provides a rationale for combinatorial strategies including dietary interventions to restrict both endogenous synthesis and exogenous lipid availability.

### Dietary interventions

*Ketogenic diets (KDs)* are among the most advanced lipid-focused dietary approaches, with established safety profiles in pediatric populations [108, 109]. KDs are characterized by high fat and very low carbohydrate content (typically a 4:1 ratio of fat (in grams) to protein plus carbohydrate (in grams)) [109], which induces a fasting-like metabolic state that reduces blood glucose and insulin levels while promoting ketone body production. These metabolic alterations enhance the anti-tumor effect of PI3K inhibitors [110] and conventional chemotherapy [111]. Strategic selection of the FA composition within a KD also promotes the accumulation of toxic SFAs in tumor tissues and may synergize with SCD inhibitors [112]. However, systemic NADPH depletion induced by KDs can impair corticosterone biosynthesis, and accelerate cachexia in preclinical models despite tumor suppression, illustrating the complex whole-body metabolic consequences of dietary interventions [113].

*Omega-3 PUFA supplementation* is increasingly evaluated in pediatric oncology due to the well-documented anti-inflammatory properties that may reduce chemotherapy-associated toxicities [114, 115]. Preclinical studies have further demonstrated direct anti-tumor activity of omega-3 PUFAs in neuroblastoma models [116]. In clinical settings, omega-3 supplementation in pediatric leukemia has yielded promising outcomes [117–119], with additional trials ongoing [NCT04209244]. While these findings suggest therapeutic potential, larger controlled trials are needed to establish efficacy across pediatric cancer subtypes.

### The road ahead for research

We would like to highlight three major obstacles impeding clinical translation of therapies targeting lipid metabolism: 1) claims of metabolic vulnerabilities based solely on pathway surrogates, 2) incomplete understanding of in-patient metabolic dependencies, and 3) metabolic heterogeneity within tumors.

In everyday practice, metabolic vulnerabilities are often inferred from surrogate measurements such as DNA or RNA-sequencing from tumor biopsies. This reflects the broad availability of these readouts in routine diagnostic workflows. While enzyme expression can inform on the potential capacity of a pathway, these methods do not actually quantify metabolic activity. Over the last decade, advances in lipidomics have increasingly overcome the long-standing analytical challenges stemming from the structural complexity of lipids [120]. We expect that the broad application of lipidomics in bulk tissues and at single-cell and spatial levels will further expand our currently limited knowledge of lipid phenotypes and metabolic heterogeneity. Complementing pool-size measurements with stable isotope tracing will enable direct interrogation of metabolic fluxes. Knowledge of lipid production and degradation rates within tumors can delineate local turnover from uptake and inform the identification of cancer-specific targets based on metabolic activity. Particularly promising are approaches that employ novel tracers or the quantification of absolute fluxes beyond measuring nutrient contributions at steady state [121], as it has recently been shown for the TCA cycle [122]. Together, lipidomics and tracing are thus uniquely suited to resolve cancer lipid metabolism.

Metabolic pathway use can differ in patients compared with preclinical models, underscoring the need for direct in-patient measurements [121]. Recent demonstrations of safe and feasible in-patient stable isotope tracing have opened new avenues for studying cancer lipid metabolism [121, 123, 124]. Remaining challenges largely come from the organizational complexity in clinical workflows: coordinating clinical-grade stable isotope availability, establishing standardized protocols for patient administration, and defining therapy-relevant readouts. For the future, we envision that tracing and lipidomics will guide clinical trials, by stratifying patients, assessing target engagement, and identifying compensatory metabolic adaptations [121].

Finally, accounting for metabolic heterogeneity will be crucial. Advanced imaging techniques are increasingly enabling direct visualization of lipid distributions within tissues [125, 126]. Beyond pool-size measurements, emerging tracing techniques enable spatial resolution at the single-cell level, and their integration with spatial transcriptomics or proteomics holds great promise for mapping cellular heterogeneity to reveal subclone-specific dependencies [127].

Together, these considerations underscore that effective lipid-targeting therapies will require a precision-medicine framework that defines metabolic dependencies in patients and accounts for metabolic heterogeneity. With continued advances in stable isotope tracing and lipidomics, the field is now well-positioned to implement these approaches in clinically relevant settings and make meaningful strides toward clinical impact.

## Conclusion

Pediatric cancers rewire lipid metabolism to gain survival advantages. Cancer cells synthesize lipids in nutrient-poor niches, scavenge them in lipid-rich environments, and rapidly activate compensatory pathways when therapeutically challenged. These adaptations enable proliferation, ferroptosis resistance and metastatic colonization, establishing lipid metabolism as a defining feature of pediatric cancer biology. This very adaptability has also significantly slowed therapeutic development. Fortunately, we now have more tools to interrogate lipid metabolism in cancer patients than ever before. We envision the therapeutic future to be based on combinatorial interventions and personalized to each patient’s metabolic fingerprint. Thus, lipid-targeting therapies are poised to contribute to the fight against childhood cancer.

## References

[1] Gröbner SN, Worst BC, Weischenfeldt J, Buchhalter I, Kleinheinz K, Rudneva VA, et al. The landscape of genomic alterations across childhood cancers. Nature. 2018;555:321–7. 29489754 10.1038/nature25480

[2] Chen X, Yang W, Roberts CWM, Zhang J. Developmental origins shape the paediatric cancer genome. Nat Rev Cancer. 2024;24:382–98. 38698126 10.1038/s41568-024-00684-9 PMC11571274

[3] Saldana-Guerrero IM, Montano-Gutierrez LF, Boswell K, Hafemeister C, Poon E, Shaw LE, et al. A human neural crest model reveals the developmental impact of neuroblastoma-associated chromosomal aberrations. Nat Commun. 2024;15:3745. 38702304 10.1038/s41467-024-47945-7 PMC11068915

[4] Cliff TS, Wu T, Boward BR, Yin A, Yin H, Glushka JN, et al. MYC controls human pluripotent stem cell fate decisions through regulation of metabolic flux. Cell Stem Cell. 2017;21:502–16.e9. 28965765 10.1016/j.stem.2017.08.018 PMC5644510

[5] Aaltonen LA, Abascal F, Abeshouse A, Aburatani H, Adams DJ, Agrawal N, et al. Pan-cancer analysis of whole genomes. Nature. 2020;578:82–93. 32025007 10.1038/s41586-020-1969-6 PMC7025898

[6] Alexandrov LB, Nik-Zainal S, Wedge DC, Aparicio SAJR, Behjati S, Biankin AV, et al. Signatures of mutational processes in human cancer. Nature. 2013;500:415–21. 23945592 10.1038/nature12477 PMC3776390

[7] Zhang L, Zhao J, Lam SM, Chen L, Gao Y, Wang W, et al. Low-input lipidomics reveals lipid metabolism remodelling during early mammalian embryo development. Nat Cell Biol. 2024;26:278–93. 38302721 10.1038/s41556-023-01341-3

[8] Gonzalez-Bohorquez D, Gallego López IM, Jaeger BN, Pfammatter S, Bowers M, Semenkovich CF, et al. FASN-dependent de novo lipogenesis is required for brain development. Proc Natl Acad Sci. 2022;119:e2112040119. 34996870 10.1073/pnas.2112040119 PMC8764667

[9] Dixon SJ, Olzmann JA. The cell biology of ferroptosis. Nat Rev Mol Cell Biol. 2024;25:424–42. 38366038 10.1038/s41580-024-00703-5 PMC12187608

[10] Yuan Y, Li P, Li J, Zhao Q, Chang Y, He X. Protein lipidation in health and disease: molecular basis, physiological function and pathological implication. Signal Transduct Target Ther. 2024;9:60. 38485938 10.1038/s41392-024-01759-7 PMC10940682

[11] Dyall SC, Balas L, Bazan NG, Brenna JT, Chiang N, da Costa Souza F, et al. Polyunsaturated fatty acids and fatty acid-derived lipid mediators: recent advances in the understanding of their biosynthesis, structures, and functions. Prog Lipid Res. 2022;86:101165. 35508275 10.1016/j.plipres.2022.101165 PMC9346631

[12] Shi X, Zheng Y, Jiang L, Zhou B, Yang W, Li L, et al. EWS-FLI1 regulates and cooperates with core regulatory circuitry in Ewing sarcoma. Nucleic Acids Res. 2020;48:11434–51. 33080033 10.1093/nar/gkaa901 PMC7672457

[13] Sabatier M, Birsen R, Lauture L, Mouche S, Angelino P, Dehairs J, et al. C/EBPα confers dependence to fatty acid anabolic pathways and vulnerability to lipid oxidative stress–induced ferroptosis in FLT3-mutant leukemia. Cancer Discov. 2023;13:1720–47. 37012202 10.1158/2159-8290.CD-22-0411

[14] Zirath H, Frenzel A, Oliynyk G, Segerström L, Westermark UK, Larsson K, et al. MYC inhibition induces metabolic changes leading to accumulation of lipid droplets in tumor cells. Proc Natl Acad Sci USA. 2013;110:10258–63. 23733953 10.1073/pnas.1222404110 PMC3690852

[15] Bhatia B, Hsieh M, Kenney AM, Nahlé Z. Mitogenic Sonic hedgehog signaling drives E2F1-dependent lipogenesis in progenitor cells and medulloblastoma. Oncogene. 2011;30:410–22. 20890301 10.1038/onc.2010.454 PMC3072890

[16] Guertin DA, Wellen KE. Acetyl-CoA metabolism in cancer. Nat Rev Cancer. 2023;23:156–72. 36658431 10.1038/s41568-022-00543-5 PMC11137663

[17] Carroll PA, Diolaiti D, McFerrin L, Gu H, Djukovic D, Du J, et al. Deregulated Myc requires MondoA/Mlx for metabolic reprogramming and tumorigenesis. Cancer Cell. 2015;27:271–85. 25640402 10.1016/j.ccell.2014.11.024 PMC4326605

[18] Guerra RM, Pagliarini DJ. Coenzyme Q biochemistry and biosynthesis. Trends Biochem. Sci. 2023;48:463–76. 36702698 10.1016/j.tibs.2022.12.006 PMC10106368

[19] Schumacher MM, DeBose-Boyd RA. Posttranslational regulation of HMG CoA reductase, the rate-limiting enzyme in synthesis of cholesterol. Annu Rev Biochem. 2021;90:659–79. 34153214 10.1146/annurev-biochem-081820-101010

[20] Wang M, Casey PJ. Protein prenylation: unique fats make their mark on biology. Nat Rev Mol Cell Biol. 2016;17:110–22. 26790532 10.1038/nrm.2015.11

[21] Huang B, Song BL, Xu C. Cholesterol metabolism in cancer: mechanisms and therapeutic opportunities. Nat Metab. 2020;2:132–41. 32694690 10.1038/s42255-020-0174-0

[22] Deshpande I, Liang J, Hedeen D, Roberts KJ, Zhang Y, Ha B, et al. Smoothened stimulation by membrane sterols drives Hedgehog pathway activity. Nature. 2019;571:284–8. 31263273 10.1038/s41586-019-1355-4 PMC6709672

[23] Tan SH, Tan TK, Yokomori R, Liao M, Huang XZ, Yeoh AEJ, et al. TAL1 hijacks MYCN enhancer that induces MYCN expression and dependence on mevalonate pathway in T-cell acute lymphoblastic leukemia. Leukemia. 2023;37:1969–81. 37591943 10.1038/s41375-023-01993-y

[24] Simopoulos AP. The importance of the ratio of omega-6/omega-3 essential fatty acids. Biomed. Pharmacother. 2002;56:365–79. 12442909 10.1016/s0753-3322(02)00253-6

[25] Bartolacci C, Andreani C, Vale G, Berto S, Melegari M, Crouch AC, et al. Targeting de novo lipogenesis and the Lands cycle induces ferroptosis in KRAS-mutant lung cancer. Nat Commun. 2022;13:4327. 35882862 10.1038/s41467-022-31963-4 PMC9325712

[26] Zadoorian A, Du X, Yang H. Lipid droplet biogenesis and functions in health and disease. Nat Rev Endocrinol. 2023;19:443–59. 37221402 10.1038/s41574-023-00845-0 PMC10204695

[27] Peng P, Chavel C, Liu W, Carlson LM, Cao S, Utley A, et al. Pro-survival signaling regulates lipophagy essential for multiple myeloma resistance to stress-induced death. Cell Rep. 2024;43. Available from: https://www.cell.com/cell-reports/abstract/S2211-1247(24)00774-5. 10.1016/j.celrep.2024.114445 PMC11318075 38968073

[28] Gryder BE, Yohe ME, Chou HC, Zhang X, Marques J, Wachtel M, et al. PAX3-FOXO1 establishes myogenic super enhancers and confers BET bromodomain vulnerability. Cancer Discov. 2017;7:884–99. 28446439 10.1158/2159-8290.CD-16-1297 PMC7802885

[29] Durbin AD, Zimmerman MW, Dharia NV, Abraham BJ, Iniguez AB, Weichert-Leahey N, et al. Selective gene dependencies in MYCN-amplified neuroblastoma include the core transcriptional regulatory circuitry. Nat Genet. 2018;50:1240–6. 30127528 10.1038/s41588-018-0191-z PMC6386470

[30] Liu M, Xia Y, Ding J, Ye B, Zhao E, Choi JH, et al. Transcriptional profiling reveals a common metabolic program in high-risk human neuroblastoma and mouse neuroblastoma sphere-forming cells. Cell Rep. 2016;17:609–23. 27705805 10.1016/j.celrep.2016.09.021 PMC5536348

[31] Gizaw NY, Kolari K, Kallio P, Alitalo K, Kivelä R Inhibiting cholesterol synthesis halts rhabdomyosarcoma growth via ER stress and cell cycle arrest. EMBO Mol Med. 2025. Available from: 10.1038/s44321-025-00336-x. 10.1038/s44321-025-00336-x PMC12686467 41249736

[32] Moreno-Smith M, Milazzo G, Tao L, Fekry B, Zhu B, Mohammad MA, et al. Restoration of the molecular clock is tumor suppressive in neuroblastoma. Nat Commun. 2021;12:4006. 34183658 10.1038/s41467-021-24196-4 PMC8238982

[33] Shimano H, Sato R. SREBP-regulated lipid metabolism: convergent physiology — divergent pathophysiology. Nat Rev Endocrinol. 2017;13:710–30. 28849786 10.1038/nrendo.2017.91

[34] Tao L, Mohammad MA, Milazzo G, Moreno-Smith M, Patel TD, Zorman B, et al. MYCN-driven fatty acid uptake is a metabolic vulnerability in neuroblastoma. Nat Commun. 2022;13:3728. 35764645 10.1038/s41467-022-31331-2 PMC9240069

[35] Lv K, Ren JG, Han X, Gui J, Gong C, Tong W. Depalmitoylation rewires FLT3-ITD signaling and exacerbates leukemia progression. Blood. 2021;138:2244–55. 34111291 10.1182/blood.2021011582 PMC8832469

[36] Choudhary C, Olsen JV, Brandts C, Cox J, Reddy PNG, Böhmer FD, et al. Mislocalized activation of oncogenic RTKs switches downstream signaling outcomes. Mol Cell. 2009;36:326–39. 19854140 10.1016/j.molcel.2009.09.019

[37] Li Y, Yang W, Patel RM, Casey EB, Denby E, Mendoza-Castrejon J, et al. FLT3ITD drives context-specific changes in cell identity and variable interferon dependence during AML initiation. Blood. 2023;141:1442–56. 36395068 10.1182/blood.2022016889 PMC10082380

[38] Park AK, Lee JY, Cheong H, Ramaswamy V, Park SH, Kool M, et al. Subgroup-specific prognostic signaling and metabolic pathways in pediatric medulloblastoma. BMC Cancer. 2019;19:571. 31185958 10.1186/s12885-019-5742-x PMC6560914

[39] Buchou C, Laud-Duval K, van der Ent W, Grossetête S, Zaidi S, Gentric G, et al. Upregulation of the mevalonate pathway through EWSR1-FLI1/EGR2 regulatory axis confers Ewing cells exquisite sensitivity to statins. Cancers. 2022;14:2327. 35565457 10.3390/cancers14092327 PMC9100622

[40] Tran GB, Ding J, Ye B, Liu M, Yu Y, Zha Y, et al. Caffeine supplementation and FOXM1 inhibition enhance the antitumor effect of statins in neuroblastoma. Cancer Res. 2023;83:2248–61. 37057874 10.1158/0008-5472.CAN-22-3450 PMC10320471

[41] Codenotti S, Asperti M, Poli M, Lorenzi L, Pietrantoni A, Cassandri M, et al. Synthetic inhibition of SREBP2 and the mevalonate pathway blocks rhabdomyosarcoma tumor growth in vitro and in vivo and promotes chemosensitization. Mol Metab. 2025;92:102085. 39706565 10.1016/j.molmet.2024.102085 PMC11750561

[42] Rashkovan M, Albero R, Gianni F, Perez-Duran P, Miller HI, Mackey AL, et al. Intracellular cholesterol pools regulate oncogenic signaling and epigenetic circuitries in early T-cell precursor acute lymphoblastic leukemia. Cancer Discov. 2022;12:856–71. 34711640 10.1158/2159-8290.CD-21-0551 PMC8904296

[43] Zhuang L, Lin J, Lu ML, Solomon KR, Freeman MR. Cholesterol-rich lipid rafts mediate Akt-regulated survival in prostate cancer cells1. Cancer Res. 2002;62:2227–31. 11956073

[44] Raleigh DR, Sever N, Choksi PK, Sigg MA, Hines KM, Thompson BM, et al. Cilia-associated oxysterols activate smoothened. Mol Cell. 2018;72:316–27.e5. 30340023 10.1016/j.molcel.2018.08.034 PMC6503851

[45] Daggubati V, Hochstelter J, Bommireddy A, Choudhury A, Krup AL, Kaur P, et al. Smoothened-activating lipids drive resistance to CDK4/6 inhibition in Hedgehog-associated medulloblastoma cells and preclinical models. J Clin Investig. 2021;131. Available from: https://www.jci.org/articles/view/141171. 10.1172/JCI141171 PMC7954583 33476305

[46] Gordon RE, Zhang L, Peri S, Kuo YM, Du F, Egleston BL, et al. Statins synergize with Hedgehog Pathway inhibitors for treatment of medulloblastoma. Clin Cancer Res. 2018;24:1375–88. 29437795 10.1158/1078-0432.CCR-17-2923 PMC5856627

[47] Ma J, Wang S, Zhang P, Zheng S, Li X, Li J, et al. Emerging roles for fatty acid oxidation in cancer. Genes Dis. 2025;12:101491. 40290117 10.1016/j.gendis.2024.101491 PMC12022645

[48] Savino AM, Fernandes SI, Olivares O, Zemlyansky A, Cousins A, Markert EK, et al. Metabolic adaptation of acute lymphoblastic leukemia to the central nervous system microenvironment depends on stearoyl-CoA desaturase. Nat Cancer. 2020;1:998–1009. 33479702 10.1038/s43018-020-00115-2 PMC7116605

[49] Cousins A, Olivares O, Markert E, Manoharan A, Bubnova X, Bresolin S, et al. Central nervous system involvement in childhood acute lymphoblastic leukemia is linked to upregulation of cholesterol biosynthetic pathways. Leukemia. 2022;36:2903–7. 36289348 10.1038/s41375-022-01722-x PMC9712090

[50] Ricciardi MR, Mirabilii S, Allegretti M, Licchetta R, Calarco A, Torrisi MR, et al. Targeting the leukemia cell metabolism by the CPT1a inhibition: functional preclinical effects in leukemias. Blood. 2015;126:1925–9. 26276667 10.1182/blood-2014-12-617498

[51] Tcheng M, Roma A, Ahmed N, Smith RW, Jayanth P, Minden MD, et al. Very long chain fatty acid metabolism is required in acute myeloid leukemia. Blood. 2021;137:3518–32. 33720355 10.1182/blood.2020008551 PMC8225921

[52] Miyagaki S, Kikuchi K, Mori J, Lopaschuk GD, Iehara T, Hosoi H. Inhibition of lipid metabolism exerts antitumor effects on rhabdomyosarcoma. Cancer Med. 2021;10:6442–55. 34472721 10.1002/cam4.4185 PMC8446407

[53] Yang M, Wei R, Zhang S, Hu S, Liang X, Yang Z, et al. NSUN2 promotes osteosarcoma progression by enhancing the stability of FABP5 mRNA via m5C methylation. Cell Death Dis. 2023;14:125. 36792587 10.1038/s41419-023-05646-x PMC9932088

[54] Oliynyk G, Ruiz-Pérez MV, Sainero-Alcolado L, Dzieran J, Zirath H, Gallart-Ayala H, et al. MYCN-enhanced oxidative and glycolytic metabolism reveals vulnerabilities for targeting neuroblastoma. iScience. 2019;21:188–204. 31670074 10.1016/j.isci.2019.10.020 PMC6889365

[55] Zhang J, Xu S, Fang H, Wu D, Ouyang C, Shi Y, et al. CAMKIIδ reinforces lipid metabolism and promotes the development of B cell lymphoma. Adv Sci. 2025;12:2409513. 10.1002/advs.202409513 PMC11905072 39840457

[56] Eguchi J, Wang X, Yu S, Kershaw EE, Chiu PC, Dushay J, et al. Transcriptional control of adipose lipid handling by IRF4. Cell Metab. 2011;13:249–59. 21356515 10.1016/j.cmet.2011.02.005 PMC3063358

[57] Koppenol WH, Bounds PL, Dang CV. Otto Warburg’s contributions to current concepts of cancer metabolism. Nat Rev Cancer. 2011;11:325–37. 21508971 10.1038/nrc3038

[58] Wang K, Xiao Y, Wan J, Chu Y, Zheng R, Lv F, et al. HADHA-mediated regulation of JAK/STAT3 signaling in glioblastoma: a metabolic-epigenetic axis. Cell Death Discov. 2025;11:361. 40750765 10.1038/s41420-025-02660-0 PMC12316893

[59] Mack SC, Witt H, Piro RM, Gu L, Zuyderduyn S, Stütz AM, et al. Epigenomic alterations define lethal CIMP-positive ependymomas of infancy. Nature. 2014;506:445–50. 24553142 10.1038/nature13108 PMC4174313

[60] Michealraj KA, Kumar SA, Kim LJY, Cavalli FMG, Przelicki D, Wojcik JB, et al. Metabolic regulation of the epigenome drives lethal infantile ependymoma. Cell. 2020;181:1329–45.e24. 32445698 10.1016/j.cell.2020.04.047 PMC10782558

[61] Ju HQ, Lin JF, Tian T, Xie D, Xu RH. NADPH homeostasis in cancer: functions, mechanisms and therapeutic implications. Signal Transduct Target Ther. 2020;5:231. 33028807 10.1038/s41392-020-00326-0 PMC7542157

[62] Jeon SM, Chandel NS, Hay N. AMPK regulates NADPH homeostasis to promote tumour cell survival during energy stress. Nature. 2012;485:661–5. 22660331 10.1038/nature11066 PMC3607316

[63] Pike LS, Smift AL, Croteau NJ, Ferrick DA, Wu M. Inhibition of fatty acid oxidation by etomoxir impairs NADPH production and increases reactive oxygen species resulting in ATP depletion and cell death in human glioblastoma cells. Biochim. et Biophys. Acta (BBA) Bioenerg. 2011;1807:726–34. 10.1016/j.bbabio.2010.10.022 21692241

[64] Hui S, Cowan AJ, Zeng X, Yang L, TeSlaa T, Li X, et al. Quantitative fluxomics of circulating metabolites. Cell Metab. 2020;32:676–88.e4. 32791100 10.1016/j.cmet.2020.07.013 PMC7544659

[65] Wenzel SE, Tyurina YY, Zhao J, St Croix CM, Dar HH, Mao G, et al. PEBP1 wardens ferroptosis by enabling lipoxygenase generation of lipid death signals. Cell. 2017;171:628–41.e26. 29053969 10.1016/j.cell.2017.09.044 PMC5683852

[66] Alborzinia H, Flórez AF, Kreth S, Brückner LM, Yildiz U, Gartlgruber M, et al. MYCN mediates cysteine addiction and sensitizes neuroblastoma to ferroptosis. Nat Cancer. 2022;3:471–85. 35484422 10.1038/s43018-022-00355-4 PMC9050595

[67] Guo W, Wang X, Lu B, Yu J, Xu M, Huang R, et al. Super-enhancer-driven MLX mediates redox balance maintenance via SLC7A11 in osteosarcoma. Cell Death Dis. 2023;14:439. 37460542 10.1038/s41419-023-05966-y PMC10352384

[68] Pontel LB, Bueno-Costa A, Morellato AE, Carvalho Santos J, Roué G, Esteller M. Acute lymphoblastic leukemia necessitates GSH-dependent ferroptosis defenses to overcome FSP1-epigenetic silencing. Redox Biol. 2022;55:102408. 35944469 10.1016/j.redox.2022.102408 PMC9364119

[69] Lalonde ME, Sasseville M, Gélinas AM, Milanese JS, Béland K, Drouin S, et al. Genome-wide CRISPR screens identify ferroptosis as a novel therapeutic vulnerability in acute lymphoblastic leukemia. Haematologica. 2023;108:382–93. 36134452 10.3324/haematol.2022.280786 PMC9890019

[70] Floros KV, Cai J, Jacob S, Kurupi R, Fairchild CK, Shende M, et al. MYCN-amplified neuroblastoma is addicted to iron and vulnerable to inhibition of the system Xc-/glutathione axis. Cancer Res. 2021;81:1896–908. 33483374 10.1158/0008-5472.CAN-20-1641 PMC9281612

[71] Lu Y, Yang Q, Su Y, Ji Y, Li G, Yang X, et al. MYCN mediates TFRC-dependent ferroptosis and reveals vulnerabilities in neuroblastoma. Cell Death Dis. 2021;12:511. 34011924 10.1038/s41419-021-03790-w PMC8134466

[72] Garcia-Bermudez J, Baudrier L, Bayraktar EC, Shen Y, La K, Guarecuco R, et al. Squalene accumulation in cholesterol auxotrophic lymphomas prevents oxidative cell death. Nature. 2019;567:118–22. 30760928 10.1038/s41586-019-0945-5 PMC6405297

[73] Freitas FP, Alborzinia H, dos Santos AF, Nepachalovich P, Pedrera L, Zilka O, et al. 7-Dehydrocholesterol is an endogenous suppressor of ferroptosis. Nature. 2024;626:401–10. 38297129 10.1038/s41586-023-06878-9

[74] Li Y, Ran Q, Duan Q, Jin J, Wang Y, Yu L, et al. 7-Dehydrocholesterol dictates ferroptosis sensitivity. Nature. 2024;626:411–8. 38297130 10.1038/s41586-023-06983-9 PMC11298758

[75] Mishima E, Ito J, Wu Z, Nakamura T, Wahida A, Doll S, et al. A non-canonical vitamin K cycle is a potent ferroptosis suppressor. Nature. 2022;608:778–83. 35922516 10.1038/s41586-022-05022-3 PMC9402432

[76] Bersuker K, Hendricks JM, Li Z, Magtanong L, Ford B, Tang PH, et al. The CoQ oxidoreductase FSP1 acts parallel to GPX4 to inhibit ferroptosis. Nature. 2019;575:688–92. 31634900 10.1038/s41586-019-1705-2 PMC6883167

[77] Doll S, Freitas FP, Shah R, Aldrovandi M, da Silva MC, Ingold I, et al. FSP1 is a glutathione-independent ferroptosis suppressor. Nature. 2019;575:693–8. 31634899 10.1038/s41586-019-1707-0

[78] Warner GJ, Berry MJ, Moustafa ME, Carlson BA, Hatfield DL, Faust JR. Inhibition of selenoprotein synthesis by selenocysteine tRNA[Ser]Sec lacking isopentenyladenosine*. J Biol Chem. 2000;275:28110–9. 10821829 10.1074/jbc.M001280200

[79] Moustafa ME, Carlson BA, El-Saadani MA, Kryukov GV, Sun QA, Harney JW, et al. Selective inhibition of selenocysteine tRNA maturation and selenoprotein synthesis in transgenic mice expressing isopentenyladenosine-deficient selenocysteine tRNA. Mol Cell Biol. 2001;21:3840–52. 11340175 10.1128/MCB.21.11.3840-3852.2001 PMC87048

[80] Magtanong L, Ko PJ, To M, Cao JY, Forcina GC, Tarangelo A, et al. Exogenous monounsaturated fatty acids promote a ferroptosis-resistant cell state. Cell Chem Biol. 2019;26:420–32.e9. 30686757 10.1016/j.chembiol.2018.11.016 PMC6430697

[81] Koeken I, Walravens M, Fernández-Acosta R, Van Hoyweghen R, Vintea I, Kong Y, et al. Dual lipid modulation overcomes ferroptosis resistance in high-risk neuroblastoma. Cell Death Differ. 2025;32:1–11. 10.1038/s41418-025-01623-3 PMC13156318 41299087

[82] Doll S, Proneth B, Tyurina YY, Panzilius E, Kobayashi S, Ingold I, et al. ACSL4 dictates ferroptosis sensitivity by shaping cellular lipid composition. Nat Chem Biol. 2017;13:91–8. 27842070 10.1038/nchembio.2239 PMC5610546

[83] Wang Y, Hu M, Cao J, Wang F, Han JR, Wu TW, et al. ACSL4 and polyunsaturated lipids support metastatic extravasation and colonization. Cell. 2025;188:412–29.e27. 39591965 10.1016/j.cell.2024.10.047

[84] Qiu Y, Wang X, Sun Y, Jin T, Tang R, Zhou X, et al. ACSL4-mediated membrane phospholipid remodeling induces integrin β1 activation to facilitate triple-negative breast cancer metastasis. Cancer Res. 2024;84:1856–71. 38471082 10.1158/0008-5472.CAN-23-2491 PMC11148537

[85] Dang W, Li Q, Wang X. ACSL4 promotes the formation of the proliferative subtype in hepatoblastoma. BMC Cancer. 2025;25:191. 39901207 10.1186/s12885-025-13592-4 PMC11789379

[86] Li X, Chen Q, Zhao D, Tan J, Liao R, Gu Y, et al. ACSL4 accelerates osteosarcoma progression via modulating TGF-β/Smad2 signaling pathway. Mol Cell Biochem. 2025;480:549–62. 38564125 10.1007/s11010-024-04975-5 PMC11695466

[87] Kumar B, Orellana M, Brooks J, Madabushi SS, Vishwasrao P, Parra LE, et al. Exosome-driven lipolysis and bone marrow niche remodeling support leukemia expansion. Haematologica. 2020;106:1484–8. 33054109 10.3324/haematol.2019.246058 PMC8094089

[88] Shafat MS, Oellerich T, Mohr S, Robinson SD, Edwards DR, Marlein CR, et al. Leukemic blasts program bone marrow adipocytes to generate a protumoral microenvironment. Blood. 2017;129:1320–32. 28049638 10.1182/blood-2016-08-734798

[89] Tucci J, Chen T, Margulis K, Orgel E, Paszkiewicz RL, Cohen MD, et al. Adipocytes provide fatty acids to acute lymphoblastic leukemia cells. Front Oncol. 2021;11. Available from: https://www.frontiersin.org/journals/oncology/articles/10.3389/fonc.2021.665763/full. 10.3389/fonc.2021.665763 PMC8100891 33968771

[90] Lee CK, Jeong SH, Jang C, Bae H, Kim YH, Park I, et al. Tumor metastasis to lymph nodes requires YAP-dependent metabolic adaptation. Science. 2019;363:644–9. 30733421 10.1126/science.aav0173

[91] Ubellacker JM, Tasdogan A, Ramesh V, Shen B, Mitchell EC, Martin-Sandoval MS, et al. Lymph protects metastasizing melanoma cells from ferroptosis. Nature. 2020;585:113–8. 32814895 10.1038/s41586-020-2623-z PMC7484468

[92] Kramer K, Kushner B, Heller G, Cheung NKV. Neuroblastoma metastatic to the central nervous system. Cancer. 2001;91:1510–9. 11301399

[93] HR Jin, Wang J, Wang ZJ, Xi MJ, Xia BH, Deng K, et al. Lipid metabolic reprogramming in tumor microenvironment: from mechanisms to therapeutics. J Hematol Oncol. 2023;16:103. 37700339 10.1186/s13045-023-01498-2 PMC10498649

[94] Noureddine N, Hartling I, Wawrzyniak P, Srikanthan P, Lou PH, Lucchinetti E, et al. Lipid emulsion rich in n–3 polyunsaturated fatty acids elicits a pro-resolution lipid mediator profile in mouse tissues and in human immune cells. Am J Clin Nutr. 2022;116:786–97. 35849016 10.1093/ajcn/nqac131

[95] Ruiz-Pérez MV, Sainero-Alcolado L, Oliynyk G, Matuschek I, Balboni N, Ubhayasekera SJKA, et al. Inhibition of fatty acid synthesis induces differentiation and reduces tumor burden in childhood neuroblastoma. iScience. 2021;24. Available from: https://www.cell.com/iscience/abstract/S2589-0042(21)00096-1. 10.1016/j.isci.2021.102128 PMC7895756 33659885

[96] Kelly W, Diaz Duque AE, Michalek J, Konkel B, Caflisch L, Chen Y, et al. Phase II investigation of TVB-2640 (Denifanstat) with bevacizumab in patients with first relapse high-grade astrocytoma. Clin Cancer Res. 2023;29:2419–25. 37093199 10.1158/1078-0432.CCR-22-2807 PMC10320469

[97] Dembitz V, Lawson H, Burt R, Natani S, Philippe C, James SC, et al. Stearoyl-CoA desaturase inhibition is toxic to acute myeloid leukemia displaying high levels of the de novo fatty acid biosynthesis and desaturation. Leukemia. 2024;38:2395–409. 39187579 10.1038/s41375-024-02390-9 PMC11518998

[98] Southam AD, Khanim FL, Hayden RE, Constantinou JK, Koczula KM, Michell RH, et al. Drug redeployment to kill leukemia and lymphoma cells by disrupting SCD1-mediated synthesis of monounsaturated fatty acids. Cancer Res. 2015;75:2530–40. 25943877 10.1158/0008-5472.CAN-15-0202

[99] Kirad S, Puri S, Deepa PR, Sankaranarayanan M. An insight into advances and challenges in the development of potential stearoyl Co-A desaturase 1 inhibitors. RSC Adv. 2024;14:30487–517. 39318456 10.1039/d4ra06237j PMC11421311

[100] Schug ZT, Peck B, Jones DT, Zhang Q, Grosskurth S, Alam IS, et al. Acetyl-CoA synthetase 2 promotes acetate utilization and maintains cancer cell growth under metabolic stress. Cancer Cell. 2015;27:57–71. 25584894 10.1016/j.ccell.2014.12.002 PMC4297291

[101] Comer C, Cotton K, Edwards C, Dai X, Badodi S, Buccafusca R, et al. Simvastatin suppresses spinal cord metastasis of medulloblastoma at clinically significant doses. Cell Death Dis. 2025;16:527. 40664675 10.1038/s41419-025-07829-0 PMC12263873

[102] Cash T, Jonus HC, Tsvetkova M, Beumer JH, Sadanand A, Lee JY, et al. A phase 1 study of simvastatin in combination with topotecan and cyclophosphamide in pediatric patients with relapsed and/or refractory solid and CNS tumors. Pediatr Blood Cancer. 2023;70:e30405. 37158620 10.1002/pbc.30405 PMC11225565

[103] Rodríguez-Rodríguez R, Baena M, Zagmutt S, Paraiso WK, Reguera AC, Fadó R, et al. International Union of Basic and Clinical Pharmacology. CXIX. Fundamental insights and clinical relevance regarding the carnitine palmitoyltransferase family of enzymes. Pharmacol Rev. 2025;77:100051. 40106976 10.1016/j.pharmr.2025.100051

[104] Mañas A, Seger A, Adamska A, Smyrilli K, Siaw JT, Radke K, et al. Targeted ferroptosis induction enhances chemotherapy efficacy in chemoresistant neuroblastoma. npj Precis Onc. 2025;9:311. 10.1038/s41698-025-01090-6 PMC12441132 40957885

[105] Villablanca JG, Volchenboum SL, Cho H, Kang MH, Cohn SL, Anderson CP, et al. A phase I new approaches to neuroblastoma therapy study of buthionine sulfoximine and melphalan with autologous stem cells for recurrent/refractory high-risk neuroblastoma. Pediatr Blood Cancer. 2016;63:1349–56. 27092812 10.1002/pbc.25994 PMC8992729

[106] Wiernicki B, Maschalidi S, Pinney J, Adjemian S, Vanden Berghe T, Ravichandran KS, et al. Cancer cells dying from ferroptosis impede dendritic cell-mediated anti-tumor immunity. Nat Commun. 2022;13:3676. 35760796 10.1038/s41467-022-31218-2 PMC9237053

[107] Xu S, Chaudhary O, Rodríguez-Morales P, Sun X, Chen D, Zappasodi R, et al. Uptake of oxidized lipids by the scavenger receptor CD36 promotes lipid peroxidation and dysfunction in CD8+ T cells in tumors. Immunity. 2021;54:1561–77.e7. 34102100 10.1016/j.immuni.2021.05.003 PMC9273026

[108] AlMutairi H, Mccullough F, Siddiqui K, Ghemlas I, AlHarbi M, Grundy R, et al. Safety, feasibility, and effectiveness of ketogenic diet in pediatric patients with brain tumors: a systematic review. J Nutr Metab. 2025;2025:7935879. 40134818 10.1155/jnme/7935879 PMC11936527

[109] Hassan AM, Keene DL, Whiting SE, Jacob PJ, Champagne JR, Humphreys P. Ketogenic diet in the treatment of refractory epilepsy in childhood. Pediatr Neurol. 1999;21:548–52. 10465141 10.1016/s0887-8994(99)00045-4

[110] Hopkins BD, Pauli C, Du X, Wang DG, Li X, Wu D, et al. Suppression of insulin feedback enhances the efficacy of PI3K inhibitors. Nature. 2018;560:499–503. 30051890 10.1038/s41586-018-0343-4 PMC6197057

[111] Morscher RJ, Aminzadeh-Gohari S, Hauser-Kronberger C, Feichtinger RG, Sperl W, Kofler B. Combination of metronomic cyclophosphamide and dietary intervention inhibits neuroblastoma growth in a CD1-nu mouse model. Oncotarget. 2016;7:17060–73. 26959744 10.18632/oncotarget.7929 PMC4941371

[112] Lien EC, Westermark AM, Zhang Y, Yuan C, Li Z, Lau AN, et al. Low glycaemic diets alter lipid metabolism to influence tumour growth. Nature. 2021;599:302–7. 34671163 10.1038/s41586-021-04049-2 PMC8628459

[113] Ferrer M, Mourikis N, Davidson EE, Kleeman SO, Zaccaria M, Habel J, et al. Ketogenic diet promotes tumor ferroptosis but induces relative corticosterone deficiency that accelerates cachexia. Cell Metab. 2023;35:1147–62.e7. 37311455 10.1016/j.cmet.2023.05.008 PMC11037504

[114] Maldonado-Salinas R, Caballero-Salazar S, Castillejos-López M, Aquino-Gálvez A, Velasco-Hidalgo L, García-Guzmán A, et al. Omega-3 fatty acids and chemotherapy-induced toxicities: mechanisms and emerging evidence with a pediatric focus. Nutr Metab. 2025;22:150. 10.1186/s12986-025-01044-6 PMC12673735 41331489

[115] Mora J, Castañeda A, Gorostegui M, Santa-María V, Garraus M, Muñoz JP, et al. Naxitamab combined with granulocyte-macrophage colony-stimulating factor as consolidation for high-risk neuroblastoma patients in complete remission. Pediatr Blood Cancer. 2021;68:e29121. 34022112 10.1002/pbc.29121

[116] Gleissman H, Segerström L, Hamberg M, Ponthan F, Lindskog M, Johnsen JI, et al. Omega-3 fatty acid supplementation delays the progression of neuroblastoma in vivo. Int J Cancer. 2011;128:1703–11. 20499314 10.1002/ijc.25473

[117] Barbosa-Cortés L, Martínez-Vieyra X, Mejía-Aranguré JM, López-Alarcón M, Martin-Trejo J, Delgadillo-Portillo S, et al. Pilot study on the effect of supplementation with long-chain ω-3 polyunsaturated fatty acids on body composition in children with acute lymphoblastic leukemia: randomized clinical trial. Clin Nutr. 2023;42:1759–69. 37549598 10.1016/j.clnu.2023.06.022

[118] Zaid ZA, Shahar S, Jamal ARA, Yusof NAM. Fish oil supplementation is beneficial on caloric intake, appetite and mid upper arm muscle circumference in children with leukaemia. Asia Pac J Clin Nutr. 2012;21:502–10. 23017308

[119] Laumann RD, Iversen T, Mogensen PR, Lauritzen L, Mølgaard C, Frandsen TL. Effect of fish oil supplementation on hyperlipidemia during childhood acute lymphoblastic leukemia treatment – a pilot study. Nutr Cancer. 2021;73:1816–20. 32791015 10.1080/01635581.2020.1803934

[120] Salvador AF, Shyu CR, Parks EJ. Measurement of lipid flux to advance translational research: evolution of classic methods to the future of precision health. Exp Mol Med. 2022;54:1348–53. 36075949 10.1038/s12276-022-00838-5 PMC9534914

[121] Bartman CR, Faubert B, Rabinowitz JD, DeBerardinis RJ. Metabolic pathway analysis using stable isotopes in patients with cancer. Nat Rev Cancer. 2023;23:863–78. 37907620 10.1038/s41568-023-00632-z PMC11161207

[122] Bartman CR, Weilandt DR, Shen Y, Lee WD, Han Y, TeSlaa T, et al. Slow TCA flux and ATP production in primary solid tumours but not metastases. Nature. 2023;614:349–57. 36725930 10.1038/s41586-022-05661-6 PMC10288502

[123] Umpleby AM. Hormone Measurement Guidelines: tracing lipid metabolism: the value of stable isotopes. J Endocrinol. 2015;226:G1–10. 26047888 10.1530/JOE-14-0610

[124] Johnston K, Pachnis P, Tasdogan A, Faubert B, Zacharias LG, Vu HS, et al. Isotope tracing reveals glycolysis and oxidative metabolism in childhood tumors of multiple histologies. Med. 2021;2:395–410.e4. 33860280 10.1016/j.medj.2021.01.002 PMC8045768

[125] Samarah LZ, Zheng C, Xing X, Lee WD, Afriat A, Chitra U, et al. Spatial metabolic gradients in the liver and small intestine. Nature. 2025;648:182–90. 41094143 10.1038/s41586-025-09616-5 PMC12675281

[126] Pu S, Dou P, Xu T, Wei C, Li Y, Zhou L, et al. Spatial metabolomics strategy reveals heterogeneity of prostate cancer based on multi-platform imaging and laser microdissection-gas chromatography-tandem mass spectrometry. Talanta. 2026;298:128921. 41043271 10.1016/j.talanta.2025.128921

[127] Buglakova E, Ekelöf M, Schwaiger-Haber M, Schlicker L, Molenaar MR, Shahraz M, et al. Spatial single-cell isotope tracing reveals heterogeneity of de novo fatty acid synthesis in cancer. Nat Metab. 2024;6:1695–711. 39251875 10.1038/s42255-024-01118-4 PMC11422168

